# Development and Validation of a Risk Assessment Tool for Gaming Disorder in China: The Gaming Hazard Assessment Scale

**DOI:** 10.3389/fpubh.2022.870358

**Published:** 2022-04-11

**Authors:** Ying Tang, Zhenjiang Liao, Shucai Huang, Jingyue Hao, Qiuping Huang, Xinxin Chen, Shuhong Lin, YiFan Li, Jing Qi, Hongxian Shen

**Affiliations:** ^1^National Clinical Research Center for Mental Disorders, Department of Psychiatry, The Second Xiangya Hospital of Central South University, Changsha, China; ^2^Hunan Key Laboratory of Psychiatry and Mental Health, Hunan Medical Center for Mental Health, Chinese National Technology Institute on Mental Disorders, Institute of Mental Health of Central South University, Changsha, China; ^3^Department of Psychiatry, The Fourth People's Hospital of Wuhu, Wuhu, China; ^4^Department of Psychiatry, Brain Hospital of Hunan Province, Changsha, China

**Keywords:** addiction, Chinese, gaming disorder, psychometric properties, hazard assessment

## Abstract

Despite the growing research interest in gaming disorder, risk screening tools developed specifically for the Chinese population are still lacking. This study aimed to construct a screening tool to evaluate the risk of gaming disorder (GD) development, by assessing the severity of GD symptoms among Chinese gamers, based on clinical expert interviews, structured interviews with GD patients, a background literature review, and IGD/GD criteria proposed by the DSM-5 and ICD-11. It introduced the Gaming Hazard Assessment Scale—a multidimensional GD risk screening tool—and evaluated the dimension structure, reliability, and validity of the scale among 959 Chinese gamers. A three-level structure, consisting of 18 items scored from 0 to 54, ultimately indicated satisfactory reliability, good validity, and acceptable model fit. The scale will help large-scale initial screening and early identification of patients with a high risk of GD. Further evaluation of the Gaming Hazard Assessment Scale in clinical settings is highly recommended.

## Introduction

In 2013, the American Psychiatric Association officially included Internet gaming disorder (IGD) in the fifth edition of the Diagnostic and Statistical Manual of Mental Disorders (DSM-5) (1). In 2019, the World Health Organization (WHO) proposed the inclusion of Gaming disorder (GD), as a psychiatric disorder in the category of behavioral addiction and included it in the 11th edition of the International Classification of Diseases (ICD-11) (2). IGD is characterized by gaming behavior that lasts for at least 12 months, which is accompanied by preoccupation, loss of control and tolerance, and impairment of social functioning. Data from the Cyberspace Administration of China notes, the number of game users in China has reached 665 million as of December 2020 (3), the majority of which consisted of adolescents. Considering the vulnerability of adolescents and the accompanying impairment caused by GD, problems related to high levels of gaming and internet usage are increasingly recognized as a serious public health burden across China and the whole world (4). Depending on the differences in study design, measurement methods, and study populations used in various studies, the prevalence of IGD ranged from 0.21 to 57.5% (5). Studies have shown an alarming prevalence of IGD among adolescents, especially in males (6, 7). Further, IGD is associated with multiple health issues (e.g., physical symptoms, depressive symptoms, and sleep disturbances), especially in adolescents (8, 9). Presently, the diagnosis and treatment of GD is relatively difficult to accomplish (10), so this study focused on early identification to facilitate early preventive intervention.

Although many studies have explored new identification methods [e.g., electroencephalograph, functional magnetic resonance imaging, and biomarkers (11–14)], a convenient scale with high reliability and validity, as well as wide adaptability, is more conducive to large-scale initial screening, and it can achieve an effective balance between economic and test efficiency.

Multiple diagnostic screening tools for GD exist (15). For example, the Personal Internet Gaming Disorder Evaluation-9 (PIE-9), covered both the DSM-5 and ICD-11 diagnostic criteria, and was used to screen for IGD; however, it did not include structured interviews in its development, and it excluded participants younger than 18 years old (16). Another example is the Problematic Online Gaming Questionnaire (POGQ), which did not assess functional impairment, and it was not test–retested in the Chinese population (17). Most of these previous tools or studies are based on IGD diagnostic criteria in the DSM-5; however, relatively few tools are based on the ICD-11 (18). The Internet Gaming Disorder Test (IGD-20 Test) and the Internet Gaming Disorder Scale-Short Form (IGDS9-SF), showed good psychometric properties, though they did not account for the latest conceptualization of IGD proposed by the WHO in the ICD-11 (19, 20). Therefore, these two diagnostic criteria should be integrated to further improve the psychometric evaluation of IGD. Many studies have found that the above two criteria share components of IGD features, such as preoccupation, loss of control, and prioritizing gaming over other activities (21). However, some differences are notable. First, ICD-11 no longer emphasizes withdrawal and tolerance, which is highlighted in the DSM-5 (15). Second, functional impairment is stressed in the ICD-11, which is key for differentiating between people with gaming disorder and the large proportion of individuals engaged in intense or persistent patterns of gaming (e.g., 20–30 h per week), without experiencing associated negative consequences (22). Third, for the presentation of common symptoms, the two diagnostic criteria are not exactly the same, such as the “diminished interests” in DSM-5 and “increasing priority given to gaming” in ICD-11. Considering these limitations, this study attempted to establish a GD risk assessment scale based on both the ICD-11 and DSM-5 standards, so that it can be used for the large-scale risk screening of GD. It should be emphasized that the purpose of this scale is to identify high-risk game users at an early stage, who can be intervened in time to reduce the likelihood that they will eventually develop GD, rather than a GD diagnosis. Owing to the complexity and social characteristics of behavioral addiction, clinical diagnosis may be the best method to evaluate and diagnose GD.

The purpose of this study was to use representative samples, to construct a risk assessment scale for GD with strong applicability, high reliability and validity, and easy operation based on DSM-5 and ICD-11 criteria. This scale will especially focus on the adverse consequences (e.g., significant sleep deprivation or changes in sleep patterns, physical impairment, etc.), resulting from GD, to identify gamers who are at high risk of developing GD.

## Materials and Methods

### Item Construction

First, we invited four Chinese experts majoring in addiction, to conduct in-depth individual interviews and gather the initial item pool, based on the DSM-5 and ICD-11 criteria and their clinical experience. Concurrently, item collection was conducted by two psychiatrists, based on DSM-5 criteria in outpatients who were diagnosed with IGD, with semi-structured interviews being used to compile the items. Twenty patients who met the diagnostic criteria were selected, and they were interviewed regarding issues such as preoccupation, withdrawal, and tolerance. After the interviews, the content was sorted and classified, and keywords were extracted. Next, we reviewed relevant screening tools, such as the Internet Gaming Disorder-20 Test (23). Under the guidance of experts, we used patient interviews and references to other relevant scales to finally form an initial item pool of 47 items related to GD symptoms, which covered both the DSM-5 and ICD-11 diagnostic criteria. Specifically, we constructed a multidimensional GD model. Of the 47 item questions that were compiled, 15 candidate questions that related to the DSM-5's definition of IGD were analyzed. Each candidate question fell under one of the following categories found in the DSM-5's definition: preoccupation, tolerance, withdrawal symptoms, deception, escapism, unsuccessful control, and adventure. Fourteen candidate questions, that related to the ICD-11's definition of GD, were also analyzed. Each candidate question fell under one of two categories found in ICD-11's definition: out-of-control over gaming, and the prioritization of gaming over other hobbies and daily activities. “Continued or enhanced gaming behavior despite adverse consequences/excessive gaming use despite psychosocial problems,” is a common entry in both diagnostic criteria, and it comprised 16 questions. A close relationship between peer influence and IGD has been reported in the literature (24–26), and combined with communication with clinical patients. We also added the dimension of peer influence, including “playing games alone without friends” and “can't wait to play games with friends when they are invited to play games.”

Another four experts were then invited to conduct content validity tests, to provide suggestions on the initial scale's structure and content, the suitability of each dimension, and the semantic accuracy of each item. Some items were modified or deleted according to expert opinions. Experts repeatedly reviewed all the items and confirmed that the scale had satisfactory content validity. A pretest survey was then conducted among 113 Chinese gamers, to determine the relevance of items with GD. Based on the positive feedback provided by all participants, and after obtaining the consensus of all experts, items with weak applicability, low relevance, and poor understanding were removed, and 34 items were selected for the final test in the broader target population.

### Respondents and Procedures

Sample 1 included 572 adolescents and young adults, who were recruited from three middle schools (*n* = 372) in Changsha city, Hunan Province, by cluster sampling and online surveys (*n* = 200). To increase sample size, in-person surveys and online surveys were combined. We randomly selected different quality ranking classes and then two classes were sampled from each grade level. Three hundred and seventy-two students used a pencil and paper to complete the original 34-item scale, and participants recruited online used a QR code of Questionnaire star platform to complete the same scale anonymously, in September 2021. Each participant was questioned whether they had used the game in the past 12 months before completing the scale, and those who answered “no” were excluded. They were asked to select scale items using a four-point Likert scale, that ranged from “never” to “always” (selecting 0, 1, 2, and 3 points, respectively), with a total score ranging from 0 to 54. We performed a scale structure item analysis and an exploratory factor analysis. We then recruited another 274 participants (sample 2), from a general high school and vocational high school in Kunming City, Yunnan Province, to complete a confirmatory factor analysis (CFA). Of these 274 participants, 100 completed the Internet Addiction Test (IAT), to test criterion validity and underwent a test–retest of the Gaming Hazard Assessment Scale after 2 weeks, to assess test–retest reliability. Informed consent was obtained from all the participants before the scale was completed.

### Measures and Statistical Analyses

The structural analysis was performed using exploratory factor analysis, and item analysis was used to explore the differences between subjects with high and low scores for each item, or to conduct homogeneity research between items. The homogeneity test included a reliability test to obtain the reliability index values of scales and dimensions, and a validity test to verify the level of questionnaire validity through confirmatory factor analysis. After determining the final items and dividing the dimensions, calibration and test–retests were completed on September 7, 2021, and September 22, 2021, respectively. Spearman correlation analysis was performed to evaluate the test–retest reliability of the participants who test–retested for the Gaming Hazard Assessment Scale. Criterion validity was tested using Spearman's correlation between the Gaming Hazard Assessment Scale and IAT (27). SPSS 24.0 and AMOS version 25.0 were used in this study.

## Results

### Descriptive Statistics

The mean age of the participants in sample 1 was 19.7 years (SD = 3.929 years), of which 53.3% were male (male, *n* = 305; female, *n* = 267). The age range was 15–30 years (28, 29). The mean age of the participants in sample 2 was 16.5 years (SD = 1.765 years), of which 50.7% were male (male, *n* = 139; female, *n* = 135). The age range was 14–22 years.

### Exploratory Factor Analysis

Since the dimensions of the questions were classified according to diagnostic criteria, expert opinions, and practical experience during the preparation of the questionnaire, exploratory factor analysis was used for the 34 initial questions. First, Kaiser-Meyer-Olkin (KMO) and Bartlett sphericity tests were conducted, to verify whether the sample was suitable for exploratory factor analysis. The KMO value of the 34 items was 0.963 (*p* < 0.05), which indicated that they were suitable for factor analysis. Principal component analysis and the maximum variance method were used to determine the final factors (see Table 1).

**Table 1:** Gaming Hazard Assessment Scale KMO and Bartlett's sphericity test (sample 1, *n* = 572).

**KMO**		**0.963**
Bartlett's sphericity test	χ^2^	12,558.905
	df	561
	*p*	<0.001

Exploratory factor analysis was conducted on the scale of 34 items, and the final rotation component matrix revealed that the sample data converged after five rotations. Some factor load coefficients of items were less than 0.4 in the factor, which deviated from the corresponding relationship with the factor. Therefore, deletion was considered. A total of 16 questions were deleted, for example, “play games regardless of time or place,” “I think playing games is more important than participating in other activities,” “1 day seems like a year if I don't play games,” “I feel angry or impatient when I can't play” and so on. The final 18 items were divided into three dimensions after the deviation items were deleted, and the items with high load coefficients were selected (see Table 2). Items 5–7, 9, 22, and 25 had a high load on Factor 1 (“out-of-control” dimension); items 13, 30–32, 34, and 37–38 had a high load on Factor 2 (“impairment of social functioning” dimension); and items 12, 14–16, and 18 had a high load on Factor 3 (“cognitive impairment” dimension).

**Table 2:** Items and factor loadings of the Gaming Hazard Assessment Scale based on exploratory factor analyses (sample 1, *n* = 572).

**No**.	**Factors/Items**	**Factor loadings**		
		**Factor 1**	**Factor 2**	**Factor 3**
1	I can't control how long or how often I play games	0.778		
2	I play games more or for longer than I want	0.826		
3	Once I start playing, it's hard to stop	0.770		
5	I keep playing games even if I have lost interest or feel bored with the game itself	0.615		
18	I have tried to reduce gaming time or stop playing but failed	0.638		
21	The actual gaming time is longer than promised	0.654		
9	I feel like there's no fun in life except playing games		0.604	
26	I have conflict with family members other than parents due to playing games		0.588	
27	I seldom go out because of playing games		0.590	
28	I rarely socialize with people because of playing games		0.688	
30	I have serious sleep problems (e.g., insomnia, wakefulness) caused by playing games		0.647	
33	Playing games costs a lot of money and lowers my living standards		0.697	
34	I delay important things (e.g., exams, job hunting) because of playing games		0.714	
8	I prefer playing games to participating in other entertainment activities			0.506
10	When not playing games, I'm still thinking about games or game-related things			0.695
11	I get excited when I see or hear something about games			0.829
12	I have thoughts or urges to play games when I see or hear something related to the games			0.773
14	I get irritable when I'm asked to stop the game or reduce gaming time			0.553

*Factor 1, out-of-control; Factor 2, impairment of social functioning; Factor 3, cognitive impairment*.

In general, the results obtained by the factor load rotation component matrix of the scale data, were consistent with the scale and dimensions that were divided in the research design. Additionally, the load coefficients of the corresponding items in each dimension were all >0.45, indicating that the Gaming Hazard Assessment Scale can be used for further research and analysis.

### Project Analysis

The 18 items obtained after eliminating the deviation items, were tested by an independent sample *t*-test for the high (The top 27%), and low (The bottom 27%) score groups (see Table 3). All 18 items presented significant difference, thus indicating that they should be retained.

**Table 3:** Item analysis results of the Gaming Hazard Assessment Scale (sample 1, *n* = 572).

**Sequence no**.	**Item**	**Group**		***t* (CR)**
		**High score group (*n* = 159)**	**Low score group (*n* = 172)**	
1	5	2.92 ± 0.725	1.27 ± 0.494	−24.457^***^
2	6	3.04 ± 0.754	1.35 ± 0.515	−23.870^***^
3	7	2.93 ± 0.780	1.23 ± 0.420	−24.990^***^
4	9	2.57 ± 0.868	1.23 ± 0.500	−17.375^***^
5	12	2.63 ± 0.846	1.11 ± 0.314	−21.957^***^
6	13	2.01 ± 1.019	1.05 ± 0.291	−11.850^***^
7	14	2.55 ± 0.817	1.31 ± 0.513	−16.577^***^
8	15	2.72 ± 0.865	1.34 ± 0.488	−17.963^***^
9	16	2.72 ± 0.835	1.33 ± 0.482	−18.729^***^
10	18	2.64 ± 0.881	1.20 ± 0.441	−19.069^***^
11	22	2.57 ± 0.951	1.15 ± 0.385	−18.136^***^
12	25	2.97 ± 0.799	1.24 ± 0.427	−24.822^***^
13	30	1.86 ± 0.882	1.08 ± 0.265	−11.155^***^
14	31	2.86 ± 0.910	1.16 ± 0.381	−22.522^***^
15	32	2.43 ± 1.022	1.03 ± 0.213	−17.473^***^
16	34	2.42 ± 0.996	1.12 ± 0.328	−16.113^***^
17	37	1.84 ± 0.911	1.05 ± 0.223	−11.030^***^
18	38	1.73 ± 0.985	1.01 ± 0.108	−9.497^***^

*** *p < 0.001*.

### Reliability and Inter-factor Correlation

The Gaming Hazard Assessment Scale showed high internal consistency (α = 0.938) in the study, with Cronbach's α ranging from 0.856 to 0.896 for the three factors (see Table 4). Pearson correlation coefficients showed that the three factors were significantly and positively correlated with each other (*r* = 0.924–0.944, *p* < 0.001) on the 18-item scale (see Table 5).

**Table 4:** Reliability analysis of the Gaming Hazard Assessment Scale in three dimensions (sample 1, *n* = 572).

**Factors**	**Cronbach's α**
The whole scale	0.938
Factor 1	0.896
Factor 2	0.856
Factor 3	0.859

**Table 5:** First-order convergence validity and discriminant validity table of the Gaming Hazard Assessment Scale (sample 2, *n* = 274).

**Variable**	**CR**	**aVE**	**Factor 1**	**Factor 2**	**Factor 3**
Factor 1	0.8671	0.5243	0.724		
Factor 2	0.8248	0.4046	0.865^***^	0.636	
Factor 3	0.851	0.5346	0.864^***^	0.816^***^	0.731

*** *means p < 0.001*.

### Relationship Between the Whole Scale and Dimensions

This study further examined the relationship between the whole Gaming Hazard Assessment Scale and each dimension. Through dimensionality reduction of the dimensions' data, the Pearson correlation coefficient was used to test the correlation between each dimension and the whole scale. Pearson correlation coefficients showed that the three factors were significantly and positively correlated with each other and the whole 18-item scale (*r* = 0.924–0.944, *p* < 0.001). Among these dimensions, the correlation coefficient between the “out-of-control” dimension and the whole scale was the highest, indicating that this dimension more greatly impacts the game hazard rating level.

### Confirmatory Factor Analysis

**Figure 1:**
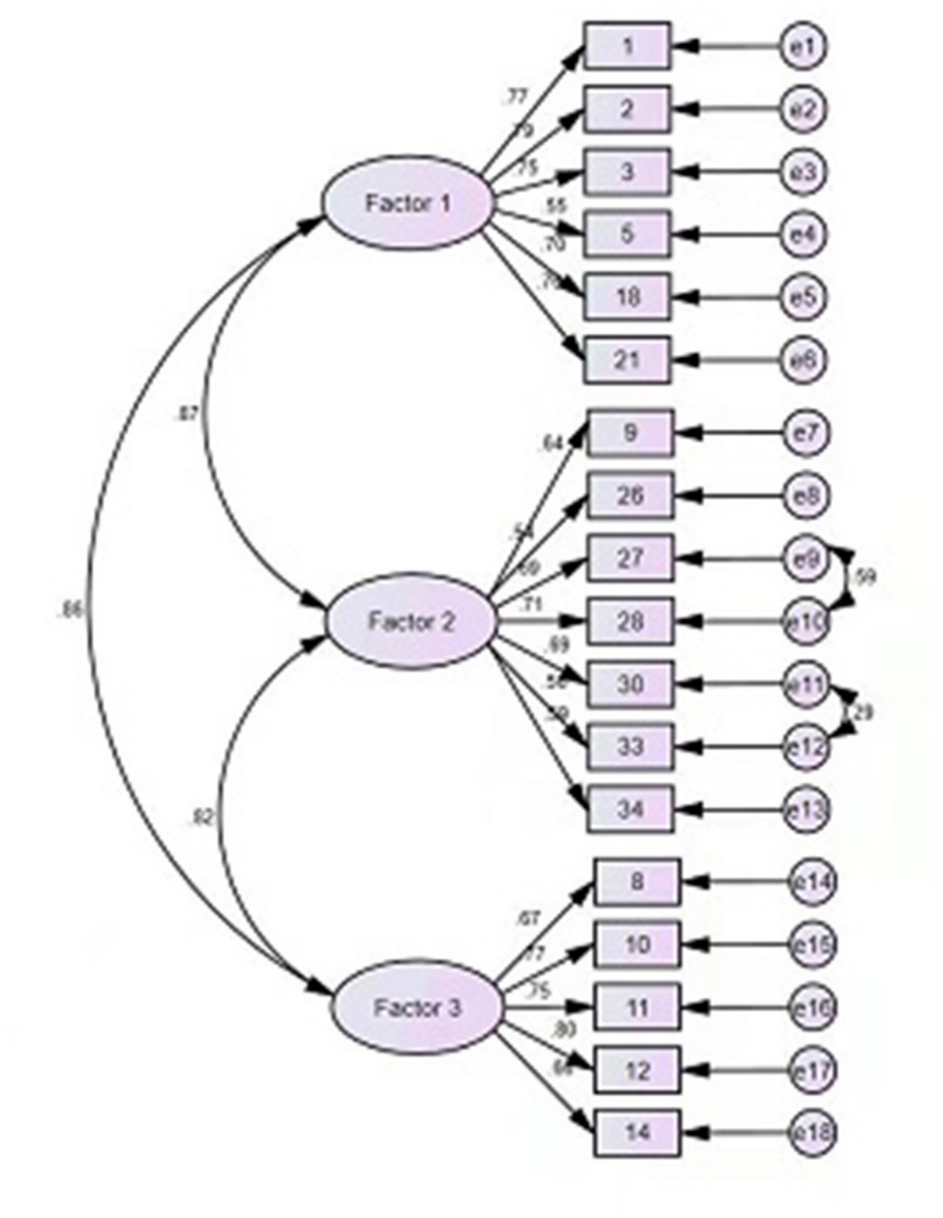
Confirmatory factor analysis.

The fit of the original three-factor model was also tested, with the results indicating that the model fit was not very good [$\chi_{\left(274\right)}^{2}$ = 3.846, *p* < 0.001, CFI = 0.857, and RMSEA = 0.102]. To optimize the model selection, model modification was considered. As all path coefficients were statistically significant, no paths were removed. We selected the Modification Index (MI), with the largest value and established a correlation between e9 and e10, e11 and e12 (see Figure 1). The results revealed that the revised model fits better than the original model [$\chi_{\left(274\right)}^{2}$ = 3.062, *p* < 0.001, CFI = 0.898, and RMSEA = 0.087]. Although the adjusted goodness-of-fit index was slightly lower, its overall model fit was better. No further corrections were made because the revised model did not result in a significant improvement.

### Convergence Validity

The evaluation criteria of convergence validity in this study were the reliability combination (CR) value and average extraction variance (aVE) value. The basic aVE value and CR value of each dimension indicate that the convergence validity of this dimension is high (see Table 5), but the square root of aVE is lower than the correlation value with other factors, so the discriminant validity of internal factors of each variable is not very good.

### Calibration Validity and Test–Retest Reliability

Twenty IAT items were used as the validity criteria of the Gaming Hazard Assessment Scale for correlation analysis. The scale was found to highly correlate with IAT scores (*r* = 0.779, *p* < 0.01), and the criterion validity was satisfied.

Two weeks later, after CFA, a total of 100 participants were test–retested, with an overall test–retest reliability of 0.535 (*p* < 0.01) (30).

## Discussion

In this study, we developed the Gaming Hazard Assessment Scale, which was developed specifically for the Chinese population, and was the first risk screening tool based on the dual diagnostic criteria of DSM-5 and ICD-11. Among the existing GD risk assessment tools, the Gaming Hazard Assessment Scale is a relatively convenient and comprehensive tool that can help clinicians effectively evaluate the risk of GD and provide targeted intervention measures. This study examined the Gaming Hazard Assessment Scale's development, as well as its psychological characteristics. The results suggest that although the Gaming Hazard Assessment Scale model fit did not reach the recommended level, it nevertheless showed satisfactory results in other aspects in the Chinese gamer sample—which supports its use as a rapid screening tool for risk assessment. Specifically, 18 items in three dimensions had factor loadings higher than the recommended level, and the overall scale and each of its factors displayed high internal consistency and acceptable test–retest reliability, and demonstrated good criterion-related and concurrent validity. This result suggests that this scale has good reliability and validity, which confirms that the Gaming Hazard Assessment Scale can be used as a relatively reliable tool for target group initial screening of patients at high risk of GD. It also provides an effective quantitative tool and evaluation standard for risk assessment of GD, to achieve early identification and intervention for GD. The relatively low test–retest reliability could be owing to memory bias caused by the time interval between tests. In addition to what has been mentioned above, the small number of participants may also be accounted for the result, which we will continue to improve in the follow-up work.

Considering that the original model's degree of fit was not satisfactory in this study, minor modifications were made to achieve sufficient model fitting (i.e., two correlations were added). One possible reason for the correlation between items 27 and 28, is that internet addiction and social phobia are considered relatively common; people with social phobia seldom go out or engage in social activities, and internet games provide them a way to reduce face-to-face contact with others while participating in game interaction (31, 32). One reason for the correlation between 30 and 33, may be that patients with severe GD can experience sleep disturbance and spend large amounts of money on games (33–35). Another possible explanation, is that players who spend money on loot boxes are more likely to play games longer on weekends than non-paying players, which indirectly leads to sleep problems (36, 37).

Although the Gaming Hazard Assessment Scale aims to identify the risk of GD development in the Chinese population, a symptomatic assessment of GD reveals only a partial picture of the clinical status of gamers with GD (38). Compared to the IGD diagnostic criteria proposed by DSM-5, the criteria proposed by ICD-11 emphasize functional impairment and consider it a core diagnostic indicator (39). The assessment of functional impairment raises the diagnostic threshold of ICD-11 for GD, avoiding the fear of pathological normal gaming behavior (40, 41), and it may thus be more advanced and applicable. The Gaming Hazard Assessment Scale displayed easy-to-operate screening applicability in assessing ICD-11's proposed GD functional impairment. Further, the three-dimensional, 18-item Gaming Hazard Assessment Scale not only included the diagnostic criteria of DSM-5, but also accounted for the clinical characteristics and game behavior patterns of ICD-11's GD diagnostic criteria, which improves the efficacy of large-scale risk screening tests for GD. Subsequent studies should consider combining the Gaming Hazard Assessment Scale with other approaches (e.g., long-term dynamic assessments of social and cognitive function, somatization disorder, and quality of life assessments), to better inform prognosis and treatment plans (42–44). Isolated studies that create multiple tools, produce an incoherent and unconvincing evidence base, so there is a need to provide a more robust theoretical and methodological basis for GD or other behavioral addictions in the population (e.g., a more integrated screening, diagnosis, and assessment system) (18). The authors intend for the development of the Gaming Hazard Assessment Scale to facilitate further empirical research on GD.

This study had some limitations and unresolved problems. First, the scale was developed specifically for Chinese participants. In this context, and considering the small sample size of the case group limited by the COVID-19 pandemic, this study can be considered a pilot study; the validity and reliability of the tool should thus be tested in future studies that involve more GD patients and other ethnic groups with different gaming cultures. Second, the test–retest reliability is not satisfactory and more than half of the study participants commenced the survey, but did not complete the test–retest. It cannot be feasibly known why the dropout rate was so high, but reasons could include the long time between tests or participants wanting to know what the survey was about and not having any intention to complete it (i.e., commencing the test out of curiosity). Whether participants who did not complete the survey differed from those who did is unclear, but this result should be accounted for when considering the study's results. Third, the discriminant validity of internal factors of each dimension is not very good, thus further studies may adjust the factor or dimension to enhance its discriminant validity. In addition, although mixed study population will benefit the availability of the scale, validity of which will also be decreased. Finally, there are some inherent disadvantages of self-reported online questionnaires, such as memory bias and social expectation bias. Future studies could attempt to add structured interviews to validate this test in a clinical setting and then estimate cutoff scores for screening purposes.

## Conclusions

The Gaming Hazard Assessment Scale is a multidimensional GD risk screening tool, which was developed specifically for the Chinese population, and was the first risk screening tool based on the dual diagnostic criteria of DSM-5 and ICD-11. This scale's development accounted for the two existing diagnostic index systems, which were characterized by ease of use, high reliability and validity, and acceptability for certain groups. Referring to IAT, the authors recommend a three categories cut-off when using this scale, which refers to score 0–18, indicating no risk, 19–36 indicating moderate risk, and 37–54 indicating high risk for GD. Considering the limitation of this study, the scale needs further validation, especially in clinical populations, to be helpful for the early identification of gamers with high-risk of GD in large scale. Reliability and validity can be further verified to expand the scope of application of the scale. Although more research is needed to confirm the Gaming Hazard Assessment Scale's ability to test in specific settings (e.g., the presence of comorbidities with other psychiatric disorders or cut off), it has great potential in terms of helping health researchers and practitioners identify potentially high-risk GD cases early. The authors also recommend that future studies should promptly apply the Gaming Hazard Assessment Scale to the clinical evaluation of GD-related risk in Chinese populations.

## Data Availability Statement

The original contributions presented in the study are included in the article/Supplementary Material, further inquiries can be directed to the corresponding author.

## Ethics Statement

Written informed consent was obtained from the individual(s), and minor(s)' legal guardian/next of kin, for the publication of any potentially identifiable images or data included in this article.

## Author Contributions

ZL, YT, and HS conceptualized and designed the research, wrote the first draft of the manuscript, and contributed to the final manuscript. QH, XC, SL, and YL prepared the assessment tools. ZL and YT performed the data collection. QH and XC undertook the statistics and analysis. All authors made substantial contributions to this study.

## Funding

This research was funded by the National Natural Science Foundation of China, grant number 81971249, the National Key R&D Program of China, grant number 2020YFC2005300, and the Natural Science Foundation of Hunan Province, grant number 2020JJ4782.

## Conflict of Interest

The authors declare that the research was conducted in the absence of any commercial or financial relationships that could be construed as a potential conflict of interest.

## Publisher's Note

All claims expressed in this article are solely those of the authors and do not necessarily represent those of their affiliated organizations, or those of the publisher, the editors and the reviewers. Any product that may be evaluated in this article, or claim that may be made by its manufacturer, is not guaranteed or endorsed by the publisher.
