# Supplementary material for: Development and Validation of a Risk Assessment Tool for Gaming Disorder in China: The Gaming Hazard Assessment Scale
Source: Front Public Health. 2022 Apr 11;10:870358. doi: 10.3389/fpubh.2022.870358 (PMC9035820; doi:10.3389/fpubh.2022.870358)
Supplement: Supplementary file 1 [file Table_1.pdf]

Appendix A

The Gaming Hazard Assessment Scale 游戏危害评估量表

In the past 12 months, how often have you experienced the following situations? (0 = never,1 = sometimes, 2 = often，3 = always) 请问在过去12个月，以下情况是否有发生在你身上? (0 = 从不，1 = 有时，2 = 常常，3 = 总是)

This scale is a screening tool rather than diagnostic tool for GD. Higher scores indicated higher risk for GD. In details, scored 0-18 indicate no risk, 19-36 indicate moderate risk and 37-54 indicate high risk. 本量表是游戏障碍的筛查工具而非诊断工具，分数越高代表患游戏障碍的风险越高,具体来说，0-18分代表无风险，19-36分代表中风险以及37-54分代表高风险。

No.	English	Chinese	Factor
1	I can't control how long or how often I play games.	我不能控制我玩游戏的时间和频率	Out of control (失控)
2	I play games more or longer than I want.	玩游戏的时间比我预计的要长	
3	Once I start playing, it’s hard to stop.	一旦开始玩游戏，很难停下	
4	I keep playing the game even if I have lost interest or feel bored with the game itself.	即使我已经失去了兴趣或对游戏本身感到厌倦，我也会继续玩游戏	
5	I have tried to reduce play time or stop playing but failed.	我试图减少游戏时间或停止游戏，但失败了	
6	The actual play time is longer than promised.	实际的游戏时间比承诺的要长	
7	I feel like there’s no fun in life except playing games.	我觉得生活中除了玩游戏就没有什么乐趣了	Impairment of social functioning（社会功能损害）
8	I have conflict with family members other than parents due to playing games.	因为玩游戏，与父母以外的家庭成员发生了冲突	
9	I seldom go out because of playing games.	因为玩游戏，我很少出去	
10	I rarely socialize with people because of playing games.	因为玩游戏，我很少与人交往	
11	I have serious sleep problems (e.g., insomnia, wakefulness) caused by playing games.	因为玩游戏，我有严重的睡眠问题(如失眠，睡不醒)	

12	Playing games costs a lot of money and lowers my living standards.	玩游戏花费了很多金钱，降低了我的生活水平	
13	I delay important things (e.g., exams, job hunting) because of playing games.	因为玩游戏，我耽误了重要的事情(比如考试，找工作)	
14	I prefer playing games to participating in other entertainment activities.	比起参加其他娱乐活动，我更喜欢玩游	Cognitive impairment（认知损害）
15	When not playing games, I'm still thinking about games or game-related things.	当我不玩游戏时，我仍然会思考游戏或与游戏相关的事情	
16	I get excited when I see or hear something about games.	当我看到或听到有关游戏的内容时，我就会感到兴奋	
17	I have thoughts or urges to play games when I see or hear something related to the games.	当我看到或听到与游戏相关的内容时，我便会产生想要玩游戏的想法或冲	
18	I get irritable when I'm asked to stop the game or reduce the time.	当我被要求停止游戏或缩短游戏时间时，我会变得烦躁	
